# Can we use human odors to diagnose malaria?

**DOI:** 10.2217/fmb-2018-0312

**Published:** 2018-12-19

**Authors:** Nina M Stanczyk, Consuelo M De Moraes, Mark C Mescher

**Affiliations:** 1 Department of Environmental Systems Science, ETH Zürich, 8092 Zürich, Switzerland

**Keywords:** diagnostics, disease biomarkers, malaria, *Plasmodium*, volatiles

After declining steadily during the early part of the twenty-first century, the worldwide incidence of malaria has recently reached a plateau [1]. Further progress toward the eradication of this devastating disease will likely require overcoming a number of serious challenges, including a need for improved methods of detecting asymptomatic infections, which currently often go undetected and untreated. Infected individuals who do not present symptoms remain capable of transmitting malaria parasites and in fact may account for 20–50% of onward transmission [1,2]. Identifying and treating these asymptomatic individuals is therefore a key goal for eradication efforts, but currently available methods for diagnostic screening of human populations, including microscopy and antibody-based rapid diagnostic tests (RDTs), have significant limitations.

A significant proportion of asymptomatic malaria cases are caused by incipient or otherwise low-level infections that are not readily detectable via microscopy [3]. RDTs may produce high rates of false negatives for these submicroscopic infections [4] and can also be unreliable for the detection of parasite species other than *Plasmodium falciparum* [5]. Furthermore, widely used RDTs based on the detection of PfHRP-2 are exhibiting increasing rates of false negatives for *P. falciparum* in some regions of Africa, India and Peru due to parasite gene deletions [5,6], raising concerns about the evolution of diagnostic resistance. A further drawback of both RDTs and microscopy-based detection methods is the need to collect blood droplets for testing, which can pose health risks in areas where blood-borne infections, such as HIV, are widespread [5]. These and other limitations of existing diagnostic methods have led to calls for the development of new detection methods that are reliable and noninvasive, and which can be easily and affordably deployed for screening of human populations [5].

Volatile compounds are an under-explored class of potential diagnostic biomarkers that could conceivably be employed for population screening to detect malarial infection. It is possible, for example, to envision the development of an easy-to-use portable device for detecting malaria-specific biomarkers that could be readily deployed in areas with limited medical infrastructure. Recently, several studies have explored the ability to detect human malaria infections via volatiles from the skin and breath, and their results suggest that volatile biomarkers may hold significant potential for the reliable detection of infected people, including those with asymptomatic infections. These promising results make a volatile diagnostic for malaria a realistic option, particularly with regard to population-wide diagnostic screening and monitoring aimed at malaria eradication.

## Potential efficacy of odor-based diagnostics for malaria

The possibility of using volatile organic compounds as a method of disease diagnosis has been under investigation for some time, with progress made in identifying volatile biomarkers for various types of cancer, lung diseases and neurogenerative diseases [7]. In some cases, unique biochemical processes associated with the diseased state may create characteristic and definitive volatile signatures. More generally, the fact that human odors are labile and highly responsive to environmental stimuli makes them potentially valuable indicators of disease status [8], perhaps especially so at the earliest stages of disease progression [9]. However, the extensive variability of human odor profiles can also pose challenges in extracting meaningful information about disease status in the presence of high levels of background variation caused by other genetic and environmental factors. It has previously been suggested that such challenges might be relatively tractable in the case of diseases caused by vector-borne pathogens, as increasing evidence suggests that such pathogens often actively manipulate host odors in ways that influence vector behavior [10]. Indeed, evidence that vector mosquito species are able to differentiate between malaria-infected and uninfected individuals based on odor [10–15] has motivated recent work on the possibility of developing a volatile-based diagnostic for malaria.

Recent studies on human malaria have examined volatiles from the skin [4,13,15] and breath [16,17] of infected volunteers, as well as volatiles collected from human blood artificially infected with *Plasmodium* parasites *in vitro* [18,19]. Each of these studies identified characteristic compounds that varied significantly between malaria-infected and uninfected hosts, strongly suggesting that malaria generates a volatile signature that might be used to detect infection via odor-based diagnostic assays. Three of these studies also employed machine learning algorithms to build predictive models for infection status [4,16,17]. This approach entails using a portion of the available data to ‘train’ the models and the remainder to test their accuracy (proportion of subjects classified correctly with respect to disease status) and sensitivity (proportion of infected subjects identified) in assessing a separate pool of individuals. The results obtained are promising, with these studies reporting an ability to identify between 70 and 100% of humans harboring *Plasmodium* parasites using models based on six or fewer compounds.

One of the two studies that analyzed breath volatiles reported that a model using four compounds (all thioethers) identified asymptomatic infections in a clinical trial with 100% sensitivity [17], while the other described a model that identified symptomatic patients at a clinic in Malawi using six chemically diverse compounds with 71% sensitivity [16]; as the clinical study had low sample size and employed artificial infections, the latter results are perhaps more indicative of the usability of breath volatiles in the field. The third study, which focused on skin volatiles collected from primary school children in Kenya, described a model that predicted infection status (across both symptomatic and symptomatic patients) with 95% sensitivity using five volatile compounds, and further reported an ability to identify submicroscopic infections with up to 20% higher sensitivity than that exhibited by RDTs in the same study [4]. This evidence that volatile diagnostics may be able to provide a higher sensitivity than currently deployed methods to low level infections suggests they could be a next step toward elimination of the parasite in human populations. Other recent studies on human skin volatiles associated with malaria infection identified changes in the emissions of several individual compounds and found that some of these elicited different responses from mosquito vectors when presented at concentrations characteristic of infected and uninfected individuals [13,15]. To the extent that changes in compounds associated with mosquito attraction may reflect ‘manipulation’ by the parasite, they should be expected to remain relatively consistent across time and space, making them promising candidates for evaluation as potential biomarkers.

Taking a broader view, it is promising that each of these recent studies – which employed diverse collection and analysis techniques and examined individuals from clinical trials as well as field populations – found differences in volatile emissions associated with malaria infection status while those employing predictive models reported an ability to identify malaria-infected individuals with relatively high sensitivity [4,13,15–17]. Volatile compounds that have so far been identified as important or predictive of malarial status in multiple studies, such as nonanal emitted from human skin and breath [4,15,16], hexanal from human skin and *Plasmodium in vitro* [4,19], toluene in human skin and *Plasmodium in vitro* [4,18] and tridecane from human breath and whole-body collections from mice [10,16], are promising candidates for use in diagnostic devices, and all of these compounds except toluene have also been shown to elicit mosquito responses [4,10]. The repeated occurrence of these compounds suggests that their relationship to infections status may be robust across disparate populations and indicates that volatile biomarkers hold genuine promise for the development of robust and practical diagnostics for malaria detection in human populations.

## Remaining challenges

Despite the promise of using volatiles as a malaria diagnostic, a number of questions remain to be addressed. In particular, two key challenges need to be overcome before volatile-based diagnostics can be considered a viable alternative to existing methods: detecting informative diagnostic compounds that are robust despite other sources of variation and developing a device capable of doing so under real-world conditions.

Overcoming the first challenge will entail the precise characterization of potential volatile biomarkers identified by recent and future studies, and determining whether they are sufficiently robust to face off broad-scale interindividual variation. Since no study to date has identified a single compound whose levels reliably predict infection status, any diagnostic method will likely have to assess the presence of a more complex disease signature involving several compounds. A further complication is that, aside from the few compounds mentioned above that have been reported from multiple populations, studies exploring the effects of malaria on human volatiles have generally identified different compounds as important predictors of infection status. In a recent field study based on skin volatiles [4], the most important predictive compounds identified, as well as model sensitivity, varied across models comparing different classes of infections status (e.g., asymptomatic and symptomatic infections), suggesting that such factors might account for some of the variation in predictors reported from other studies. Such variation might also arise from methodological differences among studies, as well as genetic variation among individuals and populations, environmental factors such as diet, or the presence of other pathogens such as HIV or helminth worms [20–22].

Human odors exhibit high intra- and interindividual variation [23], and it is not currently known whether people infected with malaria in one geographic location produce the same ‘volatile signature’ of infection as those in other areas. In addition, human malaria cases can be caused by any of five different *Plasmodium* species – as well as by mixed infections of multiple species – which might have different effects on volatile emissions. Most studies of malaria associated volatiles to date have focused on *P. falciparum*; however, a recent study [4] reported an ability to predict infection status based on skin volatiles with greater than 90% sensitivity despite a majority of subjects exhibiting mixed infections – involving *P. falciparum*, *P. malariae* and *P. ovale* – suggesting that volatile biomarkers of infection may be robust to variation in parasite species.

To date, data showing volatile shifts associated with malaria infection have come only from isolated studies carried out on individuals participating in clinical trials or from field sampling of local populations in restricted geographic areas, with these finding only a handful of characteristic compounds to be significant across multiple studies [4,13,15–17]. However, this pool of potential key volatiles provides a starting point for identifying compounds that give the highest accuracy across the broadest range of human populations and malarial species. An urgently needed next step is to carry out subsequent studies using uniform methods and analysis techniques to determine whether volatile biomarkers identified from one population are effective diagnosing infected individuals from other geographically distant populations.

The second challenge identified above entails the development of a practical diagnostic assay that can be used under field conditions. Unless further progress in refining the volatile signature of malaria is able to narrow the list of compounds required for reliable classification of disease status to one or two, this will likely require a device capable of recognizing and analyzing complex volatile signatures in the field. The technological platforms most suited to detecting multiple compounds at specific relative thresholds are biomimetic crossreactive sensor arrays, referred to in the literature as electronic noses, which are a technique currently used to detect a wide variety of diseases [7]. Sensors within these arrays are capable of recognizing compounds according to various properties (mass, electrical, electron/photon interaction), and connect to an electronic circuit able to classify samples according to patterns across compounds, matching them to previously inputted uninfected and infected ‘fingerprints’ to determine disease status in lung cancer and other diseases producing specific volatile compounds [7]. Over the last decade, biomimetic crossreactive sensor arrays devices have become more sensitive and affordable, and with time invested into method development, could become a practical platform to detect changes in volatile signatures due to malaria. Another possible avenue for the detection of malaria biomarkers involves training dogs to detect malaria-specific human odors, as they have previously been trained to do for odors associated some with some forms of cancer [24]; research into this possibility is currently underway.

## Conclusion

The development of a volatile-based diagnostic device may fulfill the need for a sensitive and noninvasive technique that could be used on a large scale in malaria-endemic countries. Although it is too early to tell if this method can be practically adapted for widespread use in the field, initial laboratory trials and localized field studies have identified characteristic volatile biomarkers that can in principle be used to predict malaria status with high sensitivity. Although many of these characteristic compounds vary between studies, there are several common compounds that provide a strong starting point for further investigation.

The two most important challenges to overcome are the effects of variation on malaria volatile signatures due to methodological, genetic and environmental factors, and the difficulty of applying volatile diagnostic methods easily and affordably in the field. A clear priority for future work is to research different populations with comparable methods and analysis to previous studies to see if the same compounds can detect a malaria volatile signature worldwide, regardless of variation in population, environment and *Plasmodium* species, or at which levels these might need to be tailored. With 2016 being the first year since 2000 in which malaria cases did not show a decline, the development of alternative technologies is a high priority to ensure that reservoirs of malaria can be fully treated to prevent ongoing transmission.
